# Heterogeneous constraint and adaptation across the malaria parasite life cycle

**DOI:** 10.1098/rspb.2025.1549

**Published:** 2025-11-12

**Authors:** Sarah A. Perkins, Daniel E. Neafsey, Angela M. Early

**Affiliations:** ^1^ Department of Immunology and Infectious Diseases, Harvard T. H. Chan School of Public Health, Boston, MA, USA; ^2^ Infectious Disease and Microbiome Program, Broad Institute, Cambridge, MA, USA

**Keywords:** selection efficacy, malaria, adaptation, complex life cycle, *Plasmodium*

## Abstract

Evolutionary forces vary across genomes, creating disparities in how traits evolve. In organisms with complex life cycles, it is unclear how intrinsic differences among discrete life stages impact evolution. Here, we look for life history-driven patterns of adaptation in *Plasmodium falciparum*, a malaria-causing parasite with a multi-stage life cycle. We posit that notable differences across the *P. falciparum* life cycle—including cell ploidy, the extent of clonal competition and the presence of transmission bottlenecks—alter the drift–selection balance acting at discrete life stages. Categorizing genes by their stages of expression, we compare patterns of between- and within-species diversity across stages. Most notably, we find signals of weaker negative selection in genes exclusively expressed in sporozoites. This matches theoretical expectations as sporozoites do not proliferate, show limited evidence of clonal competition, and pass through a strong bottleneck. We discuss how the timing of therapeutic interventions towards particular life stages might impact the rate at which parasite populations evolve resistance and consider the functional, molecular and population genetic factors that could contribute to these patterns.

## 1. Introduction

Within a species, evolution does not uniformly impact all regions of the genome. Mutation, selection, recombination, drift and gene flow may occur at different rates across loci dependent on factors ranging from chromatin accessibility [1] to tissue-specific expression [2]. Reduced substitution rates in coding regions of the genome reflect pervasive negative selection against protein variants. Strong positive selection may shift allele frequencies in only a subset of targeted genes [3]. Factors like ploidy and effective population size (*N*_e_) can also intrinsically differ across an organism’s genome. For example, in sexually reproducing diploids, adaptive substitutions accumulate more quickly in genes on sex chromosomes because the effects of recessive variants in these genes are not always masked by heterozygosity [4]. In this way, molecular, cellular and organismal characteristics shape evolutionary patterns across genomes.

The life history concept refers to major biological transitions in the life cycle of an organism, such as maturation and reproduction, which are vital to the organism’s evolutionary fitness. Life history traits, including fecundity and life span, have been demonstrated to explain levels of genetic diversity across phylogenetically diverse organisms [5]. Within-species trade-offs between life history traits are not uncommon; complex life cycles with morphologically distinct forms are hypothesized to aid adaptation at discrete life stages by decoupling selected traits [6], although our full understanding of decoupling, synergism, and antagonism across life stages is still developing [7,8]. However, with a few exceptions like diplontic plants [9] and animal gametes [10], less attention has been given to how the intrinsic characteristics of distinct life stages may themselves drive patterns of diversity within a single species’ genome. Particularly in non-metazoan organisms, complex life cycles can comprise extreme changes, not only in niche and morphology but also in ploidy and cell count. This suggests that life stages may differ both in the types of selection pressures experienced and in the efficacy of the adaptive response to those pressures.

**Figure 1.**
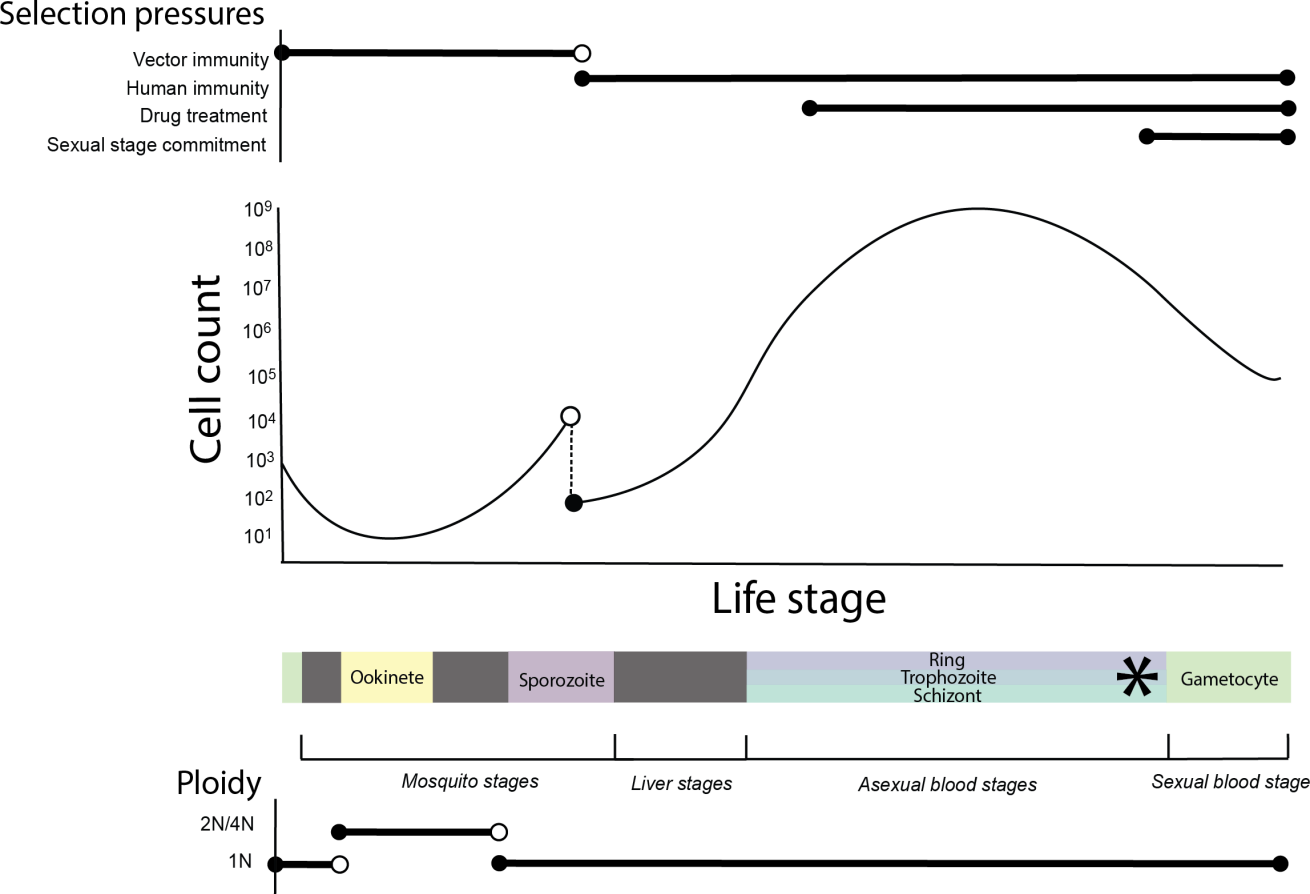
Changes in parasite cell count and ploidy coincide with diverse physiological and therapeutic selective pressures (approximated with horizontal lines) across the infectious life cycle of *P. falciparum*. Coloured boxes mark stages used in subsequent analyses and figures. The dashed line denotes a transmission bottleneck. The *P. falciparum* life cycle begins when an *Anopheles* mosquito ingests male and female gametocytes, which rapidly form gametes that fuse to produce diploid zygotes in the mosquito midgut. Zygotes undergo meiosis without cell division and develop into tetraploid ookinetes that burrow through the mosquito’s midgut epithelium. During the subsequent multinucleated oocyst stage, thousands of haploid nuclear divisions take place. Invagination of the oocyst then produces mononucleated sporozoites that migrate to the mosquito’s salivary glands [12]. Upon injection into the human host, a subset of sporozoites successfully invade the liver, where they asexually multiply 10^4^–10^5^ times before exiting into the bloodstream as merozoites [13]. Merozoites invade red blood cells and go through rounds of asexual amplification that cycle through ring, trophozoite and schizont forms. This can result in up to 10^12^ circulating parasites [14]. At each asexual replicative round, a subset of parasites instead commit to sexual development and differentiate into non-replicating male and female gametocytes (denoted by *).

Vector-transmitted parasites, like the malaria-causing apicomplexan *Plasmodium falciparum*, are excellent models for empirically studying how selection at distinct life cycle stages impacts genomic diversity. As *P. falciparum* parasites pass through the human and mosquito hosts, they undergo sophisticated stage-specific alterations in gene regulation [11] enabling invasion of and survival within diverse tissues (figure 1). While some genes are broadly expressed, others experience only a subset of the selection pressures present across the entire life cycle. Accordingly, broader expression of genes across the life cycle has been shown to correlate with greater evolutionary constraint in rodent and primate *Plasmodium* species [15].

External forces, however, are not the only factors that differ among life stages. Timing of gene expression also dictates the gene’s cellular and demographic context, which can include severe population contractions, rapid mitotic proliferation and changes in ploidy. Any of these could result in differential adaptive potential across the *P. falciparum* life cycle and impact how parasites adapt to stage-limited selection pressures.

Sexual replication of *P. falciparum* in the mosquito produces four distinct haploid genomes. As with all offspring, these siblings may differ in fitness and show differential reproductive success in subsequent rounds of sexual reproduction. However, for organisms like *Plasmodium*, differential fitness also exists between members of the sublineages that descend from each of the meiotic progeny. These sublineages acquire new mutations during mitotic divisions, and any sublineage has the potential to progress to the next round of sexual reproduction. Selection between sublineages is therefore occurring throughout the *Plasmodium* life cycle, although the efficacy of selection within each life stage is probably variable.

During the asexual blood stages, *P. falciparum* rapidly expands against a backdrop of strong selection pressures that include host immunity and drug treatments. At this stage, selection is highly efficacious and can result in drug resistance over the course of a single infection [16,17]. However, other life stages do not include such extreme opportunities for clonal interference and selection. Multiple parasite forms, including the male and female gametocyte, ookinete and sporozoite, are non-replicative and comprise lower parasite numbers. Ookinetes, oocysts and potentially sporozoites have diploid protein expression, which may mask the effects of deleterious mutations. Sporozoites and gametocytes pass through strong bottlenecks, potentially amplifying the effect of drift relative to selection. All these factors may alter the efficacy of selection on mutations whose fitness effects are limited to a specific life stage.

Previous empirical and theoretical work has touched on some but not all of these factors. Mathematical models of *P. falciparum* have demonstrated that demographic shifts associated with the human portion of the life cycle can enhance both genetic drift (due to the bottleneck into the mosquito) and selection (via clonal competition within the human host) [18]. Studies and models of other organisms with complex life cycles suggest that stage-specific selection efficacy increases with organism number and decreases with ploidy [10,19,20], although these differences have not been investigated in the context of the parasite life cycle. Understanding how the life cycle of *P. falciparum* influences adaptation will not only provide insight into a potentially important evolutionary phenomenon, but also may aid the selection of therapeutic targets—for drugs, vaccines and monoclonal antibodies—with steeper barriers to resistance evolution.

Here, we test the related hypotheses that selection efficacy and adaptation rate are heterogeneous across the *P. falciparum* life cycle. Using a single-cell sequencing dataset from the Malaria Cell Atlas (MCA) [21], we identify sets of genes expressed in single stages. We assess polymorphism in these genes using genomic variation data from four *P. falciparum* parasite populations in the MalariaGen Pf7 database [22], quantifying mean gene-level non-synonymous (*π*_NS_) and synonymous (*π*_S_) pairwise diversity, their ratio (*π*_NS_/*π*_S_), Tajima’s *D* [23] and Hudson’s *F*_ST_ [24]. We also examine between-species non-synonymous (*dN*) and synonymous (*dS*) substitution rates and their ratio (*dN/dS*) across the genome, comparing *P. falciparum* genes to orthologues in closely related species. We find that, as in other *Plasmodium* species, more broadly expressed genes are more constrained. Furthermore, we consider genes expressed only in single stages and observe signals of greater genetic drift in the sporozoite stage. We examine the distribution of fitness effects (DFE) in each stage, estimating that the sporozoite stage shows the weakest negative selection among the stages considered. We argue that it is important to extend investigations of stage-specific adaptive potential to inform the development and deployment of parasite therapeutics.

## 2. Methods

### (a) Identification of life stage-specific gene sets

To attribute *P. falciparum* gene expression to distinct life stages, we used the MCA *P. falciparum* Smart-seq2 dataset [21]. This dataset leverages high per-cell coverage single-cell sequencing technology [25] to capture gene expression in parasite cells across six life stages. Some life stages contained smaller subcategories (for example, salivary gland sporozoite and injected sporozoite); however, to create gene sets with sufficient sizes, we utilized the low-resolution life stage annotations: ookinete, sporozoite, ring, trophozoite, schizont and gametocyte.

We identified variable genes through dropout-based feature selection on normalized counts with the M3DropFeatureSelection function from M3Drop [26] (v. 1.30.0), using an FDR threshold of 0.01. This function selects genes with high residual dropout rates given their expression level, suggestive of variable expression across cell types [26]. We applied the M3DropGetMarkers function to the resulting genes to test for patterns of upregulation in single cell types, which we considered evidence for expression within that cell type. We also developed alternative gene sets with single-stage expression using previously described approaches [11,15] and a sporozoite gold standard gene set [27] (electronic supplementary material, appendix).

We excluded expected targets of immune-mediated balancing selection from both gene sets. As a proxy for antigenicity, we used reported serum antibody reactivity from a microarray-based screen of samples from a malaria-exposed cohort [28]. We removed any protein with a domain recognized by serum antibodies—producing an intensity signal at least 1 SD above the control standard—in at least 10% of the cohort (electronic supplementary material, file S1) [28]. We used ShinyGO v. 0.81 [29] to perform a gene set enrichment analysis for the resulting primary gene sets relative to the genomic background.

### (b) Characterization of breadth of gene expression across the life cycle

We counted the number of life stages each gene was expressed in to calculate expression breadth, again considering ookinete, sporozoite, ring, trophozoite, schizont and gametocyte stages. To generate binary *gene × stage* expression classifications based on MCA scRNA-seq data, we normalized the raw count matrix and discarded cells with > 150 or < 2500 transcripts. Subsequently, we set 50% as a minimum threshold for the proportion of gene-expressing cells needed to classify a gene as expressed in a given life cycle stage [11,15]. We examined the Pearson correlation between binary gene expression profiles of different stages, including genes expressed in multiple stages. Subsequently, to reduce noise from stochastic variation in background transcript detection—which could falsely increment breadth, particularly through signals of expression in smaller cell populations—we required that genes identified by this procedure demonstrate significant differential expression, via M3DropGetMarkers, uniquely in the grouped stages they were identified in compared to all other stages, grouped. This comparison was only performed for genes with breadth < 6, such that assay cells could be grouped into two groups for differential expression testing. Based on these classifications, we generated non-overlapping gene sets grouped by overall breadth of expression of each gene across the life cycle. Over half (66%) of the non-antigenic, expressed core genes could not be classified as expressed in any stages by these criteria, demonstrating limitations of this conservative filtering approach.

### (c) Population-level characterization of diversity and drift

To characterize population-wide patterns of polymorphism across life stage-specific gene sets, we used whole-genome variant calls from the MalariaGEN Pf7 data set [22]. We used data from four countries—Ghana, Tanzania, the Democratic Republic of the Congo and Cambodia—which we chose on the basis of adequate sample size (*n* ≥ 75 after filtration) and expected variation in transmission intensity.

To remove comparisons between highly related parasites, we calculated relatedness (as identity-by-descent; IBD, calculated in R via hmmIBD [30]) between all monogenomic (Fws [22] > 0.95) parasites within the same country. We formed clusters of individuals at a threshold of IBD ≥ 0.25 with the R package iGraph and retained only one sample—the sample with the highest proportion of called sites—for downstream analyses [31,32]. Mean IBD in Cambodia is high (0.12), and this filtering ensured we excluded all highly related genomes while still retaining a sufficiently large sample size. After filtering, the sample sizes from Ghana, Tanzania, the Democratic Republic of the Congo and Cambodia were 362, 136, 152 and 75, respectively (electronic supplementary material, file S2). We retained biallelic single nucleotide polymorphisms (SNPs) that passed quality filters (QC pass, VQSLOD > 2). In addition, on a per-sample basis, we retained homozygous calls with a minimum read depth of 5. We used SNPEff and SNPSift (v. 4.1) to annotate and subset synonymous (S), non-synonymous (NS) and fourfold degenerate (FFD) variants from the filtered VCF files.

For each gene across the *P. falciparum* 3D7 genome, we calculated the number and proportion of NS, S and FFD sites. We multiplied resulting counts by the variant call quality filter pass rate to adjust for missing sites (electronic supplementary material, appendix, figure S1, file S3). Using these corrected counts, we calculated gene-level NS (*π*_NS_) and S (*π*_S_) pairwise diversity, their ratio (*π*_NS_/*π*_S_) and Tajima’s *D* [23]. We expect demographic effects to manifest genome-wide and not be impacted by stage of expression and therefore assume that between-stage differences in diversity are caused by factors attributable to distinct life cycle stages. Finally, we calculated Hudson’s *F*_ST_ [24] for SNPs segregating in compared populations to examine their divergence. We calculated these measurements with scikit-allel [33].

To examine genome-wide correlations of diversity statistics with gene coding length, we used Kendall’s *τ*, a rank-based test, on untransformed data. For genes with *π*_NS_ > 0 and *π*_S_ = 0 or *dN* > 0 and *dS* = 0, we included *π*_NS_/*π*_S_ and *dN/dS* estimates as infinite and arbitrarily high values (*dN/dS* = 99, per yn00 [34], respectively. To test for stage-level differences in diversity in each population, we used linear regression to measure the association of categorical stages with gene-level diversity statistics, correcting for gene coding length. Prior to regression, we log-transformed coding length and the statistics *dN*, *dS*, *dN/dS*, *π*_NS_, *π*_S_ and *π*_NS_/*π*_S_, but not Tajima’s *D* and Hudson’s *F_ST_*, to accommodate expected log-linear relationships. In parallel with previous analyses [35], to avoid excluding zeros in log-transformed diversity statistics, we incremented all estimates by constant values set based on the minimum observed non-zero estimates of each within each population sample. We also replaced negative *F_ST_* estimates with zeros. We used Tukey tests on resulting models to test for differences in mean statistic values by stage, correcting comparisons for multiple hypothesis testing via the Benjamini–Hochberg procedure. Considering the resulting *p*-values together, we estimated the significance of meta-population differences in pairwise stage comparisons of diversity statistics via adaptively weighted Fisher (AW-Fisher) tests for combining association statistics [36]. Finally, to test for changes in *π*_NS_/*π*_S_ and *dN/dS* with breadth, we estimated partial Spearman’s rank correlation coefficients [37], controlling log-transformed estimates for log-scaled gene coding length.

### (d) Divergence calculations and DFE inference

We estimated sequence divergence between *P. falciparum* and both *P. praefalciparum* (divergence time ~ 50 000 years ago) and *P. reichenowi* (divergence time ~ 190 000 years ago) [38] using one-to-one orthologues identified with OrthoFinder [39,40]. We downloaded coding sequences from the *P. falciparum* 3D7 [41,42], *P. praefalciparum* G01 [38] and *P. reichenowi* SY57 [43] reference genomes for all genes in our life stage-specific gene sets. We aligned translated orthologues with Muscle5 implemented with default parameters in gget [44,45] and reverse-translated alignments with PAL2NAL v.14 [46]. From the resulting 4673 coding alignments, we counted NS, S and FFD variants and sites, estimated the ancestral states of NS and S variants via est-sfs [47] and estimated *dN*/*dS* via yn00 implemented in PAML v. 4.10.9 [34,48,49].

We used these divergence data with polymorphism data to perform DFE inference with DFE-alpha [50,51] and fastDFE [52]. In DFE-alpha, we used variant count and site estimates with est_alpha_omega to analyse adaptation, following deleterious DFE inference from folded site frequency spectra (SFS) under a two-epoch demographic model in est_dfe (electronic supplementary material, appendix). We used a gene-level leave-one-out approach to generate jackknifed DFE-alpha estimates of the following statistics for each gene set: the probability of fixation of a deleterious mutation (*Q*), proportion of adaptive substitution (*α*) and relative rate of adaptive divergence (*ω*). Gene set estimates were compared pairwise via the nonparametric Wilcoxon rank sum test. For fastDFE, we prepared unfolded SFS from polymorphism data polarized with inferred ancestral states. We initially used the mixed gamma and exponential DFE parameterization in fastDFE with custom *S_d_* bounds (−10^5^, −10^−20^). We used plot_nested_models in fastDFE to assess the significance of ancestral allele misidentification and beneficial fitness effects via likelihood ratio tests. We subsequently performed inference without beneficial fitness effects (constraining the probability of a beneficial mutation, *p_b_*, to 0). We compared the 95% confidence intervals of bootstrapped (*n* = 100) estimates of *α* and the mean and negative mean selection coefficients (scaled by 4*N*_e_) for beneficial (*S*_b_) and deleterious (*−S*_d_) mutations, respectively (electronic supplementary material, appendix).

## 3. Results

### (a) Single-cell sequencing captures variability in *P. falciparum* gene expression across six life cycle stages

We used single-cell Smart-seq2 *P. falciparum* data from the MCA project [21] to examine expression patterns across six *P. falciparum* life cycle stages (ookinete, sporozoite, ring, trophozoite, schizont and gametocyte; figure 1). The *P. falciparum* reference genome contains 5280 genes [41], of which a subset is reliably analysed with short-read sequence data and considered part of the ‘core’ genome [53]. 4930 of these core genes were expressed in at least 2.5% of one cell type in the assay. We excluded around 20% of detected genes with low (≤70%) variant call pass rates from population diversity statistic calculations (electronic supplementary material, figure S1A). In our evolutionary analysis, we also wished to exclude genes subject to strong immune selection. We classified 405 of the expressed core genes (8.2%) as potential antigens based on a large serum antibody reactivity screen [28] and excluded these from the main analyses (electronic supplementary material, file S1). This left us with a set of 3699 expressed non-antigenic genes for downstream characterization in parasite genomic variation data.

We classified genes by whether or not they were expressed in over 50% of the cells of each of the six life cycle stages. Examining the Pearson correlation of these binary profiles across the stages, we found negative correlations between stages in different host environments, indicating some compartmentalization of the transcriptome by stage physiology (electronic supplementary material, figure S2; ring–ookinete *r* = −0.213, *p* < 0.001; ring–gametocyte *r* = −0.178, *p <* 0.001; schizont–ookinete *r* = −0.0748, *p* < 0.01; trophozoite–ookinete *r* = −0.127, *p* < 0.001). From these profiles, we characterized the breadth of expression of genes across the six discrete life cycle stages, further applying a differential expression filter (§2). Among genes that could be classified, we estimated that most non-antigenic genes were detected in only a single stage, and very few genes were expressed across the full life cycle (*n* = 900, 402, 145, 51, 24, 6 for breadth categories 1−6; electronic supplementary material, file S3).

Next, we applied a more robust feature selection procedure to isolate genes expressed in only one life stage. We identified non-antigenic genes that were highly variable at the assay level, with significantly increased expression in a single stage. We further excluded genes with breadth estimates above 1. The resulting gene sets ranged in size from 16 to 54 genes (electronic supplementary material, figure S3 and file S4), with the fewest genes in the trophozoite category and the most genes in the gametocyte category. This finding is consistent with the latter stage encompassing morphologically distinct male and female forms, which may each demonstrate distinct levels of transcriptional activity [54]. We repeated this procedure without the feature selection step to define alternative gene sets with single-stage expression (electronic supplementary material, figure S3 and file S5).

### (b) *P. falciparum* nucleotide divergence and diversity correlate with gene length

Gene length may correlate with evolutionary rate directly or indirectly via phylogenetic depth, intronic burden [55], alternative splicing or expression level [55–57]. *P. falciparum* has unusually long genes and intergenic regions compared to other unicellular eukaryotes and protists, with a high proportion of genes over 4 kb [58,59]. We examined the effect of gene coding length genome-wide in *P. falciparum*, considering both signals of diversity and divergence. We found that *dN*, *dS* and *dN/dS* correlate positively with coding length (electronic supplementary material, figure S4A–C; Kendall’s ***τ*** = 0.412, 0.297, 0.241; all *p <* 0.001). The proportion of NS sites increased with coding length, while the proportion of S and, correspondingly, FFD sites declined (electronic supplementary material, figure S4D–F; Kendall’s *τ* = 0.260, −0.260, −0.264; all *p <* 0.001). Using signals of within-species diversity recovered from 3699 *P. falciparum* genes in a deeply sampled African population (Ghana; *n* = 362), we found that coding length positively correlates with *π*_NS_ and *π*_S_ and negatively correlates with *π*_NS_/*π*_S_ and Tajima’s *D* (electronic supplementary material, figure S5; Kendall’s *τ* = 0.191, 0.264, −0.0978, −0.605; all *p* < 0.001).

Transcript length can also influence the sensitivity of detection in RNA sequencing assays. Accordingly, we find that coding length correlates with breadth (Kendall’s *τ* = 0.200, *p <* 0.001). We also note that coding length varies in our single-stage gene sets (electronic supplementary material, figure S6C,D). Although this could represent a meaningful biological phenomenon, we aimed to test for changes in evolutionary rate independent of coding length to exclude assay-level biases as drivers of our observations. In subsequent analyses, we include coding length as a covariate to correct for this effect.

### (c) Increase in selective constraint with breadth of expression across the life cycle

We aimed to determine whether expression breadth correlates with signatures of adaptive constraint in *P. falciparum.* Based on a previous analysis of *dN/dS* in rodent *Plasmodium* spp. by Tebben *et al*. [15], we hypothesized that greater breadth of expression across the parasite life cycle would be associated with signatures of higher selective constraint, such that more broadly expressed genes would be more highly conserved both between and within species compared to more narrowly expressed genes.

We calculated a covariate-adjusted Spearman’s rank correlation coefficient that controlled for log-scaled gene coding length to analyse the genome-wide change in *P. falciparum*–*P. reichenowi dN/dS* with expression breadth, and we found a small but statistically significant negative correlation (electronic supplementary material, figure S7A; partial Spearman’s *ρ*= −0.0823, *p* = 0.00172). We found the same negative association of breadth with *dN/dS* estimates from *P. falciparum*–*P. praefalciparum* sequence comparisons (electronic supplementary material, figure S7B, partial Spearman’s *ρ* = −0.0556, *p* = 0.0418).

Next, we tested a parallel comparison of expression breadth with population-level diversity. We found a weak negative correlation of expression breadth with *π*_NS_/*π*_S_ in three of the four populations, but the pattern is not statistically significant (electronic supplementary material, figure S7C). We also found an enrichment of nuclear functions among genes expressed in more than three stages (electronic supplementary material, figure S8).

### (d) Genes expressed in single life stages do not show distinct functional enrichments

We next examined functional enrichment of the single-stage gene sets (electronic supplementary material, figure S8). These sets were limited to genes expressed in only one stage. We hypothesized that different functional enrichments could indicate functional divergence between non-overlapping gene sets, and additionally wished to determine whether any large skews existed which in and of themselves could explain divergent evolutionary patterns across life stages. We found primarily mixed enrichment signals, including enrichments of uncharacterized labels in the sporozoite, trophozoite and ookinete single-stage gene sets. We found no functional enrichments in the gametocyte single-stage gene set. In the schizont single-stage gene set, we uncovered some defined enrichments, including myristate, phosphoprotein, ANK repeat, lipid catabolic process, repeat and coiled coil. These hits may indicate a slight skew toward essential functions like lipid catabolism and toward protein–protein interactions, consistent with the intensive developmental shifts and growth occurring during this stage. However, these labels may also point to more thorough structural characterization of gene products in this set. While gaps in functional annotation across the *P. falciparum* genome [41] and the modest size of gene sets limit functional inference, we do not find clear evidence for strong functional differences between single-stage gene sets outside of a slight skew toward some metabolic and protein–protein interaction functions in the schizont set.

### (e) Substitution patterns vary across life stages

**Figure 2.**
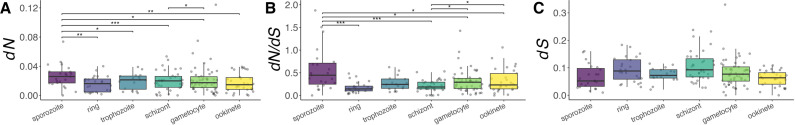
Gene-level divergence of *P. falciparum* and *P. reichenowi* orthologues by life stage (*x*-axis/colour). All estimates (A–C) were compared linearly by stage after log-transformation and correction for log-transformed coding length as described in §2. (A) BH-adjusted significant pairwise *dN* comparisons: sporozoite–ring, *p* = 0.00469; sporozoite–trophozoite, *p* = 0.0205; sporozoite–schizont, *p* = 5.39 × 10^−4^; sporozoite–gametocyte, *p* = 0.0310; sporozoite–ookinete, *p* = 0.00967; gametocyte–schizont, *p* = 0.0310. (B) BH-adjusted significant pairwise *dN/dS* comparisons: sporozoite–ring, *p* = 8.11 × 10^−4^; sporozoite–schizont, *p* = 2.70 × 10^−4^; sporozoite–gametocyte, *p* = 0.0499; sporozoite–ookinete, *p* = 0.0499, gametocyte–schizont, *p* = 0.0306, ookinete–schizont, *p* = 0.0499. (C) All BH-adjusted pairwise *dS* comparisons *p* > 0.05.

We next tested whether signatures of species-level divergence show variation across discrete life cycle stages. To do so, we used log-linear regression to compare *dN*, *dS* and *dN/dS* values for each one-to-one *P. falciparum–P. reichenowi* orthologue pair containing *P. falciparum* genes expressed in only one life cycle stage. We observed significantly higher *dN* and *dN/dS* in sporozoite-limited genes compared to genes expressed in only one of the other five stages, excluding the trophozoite stage for *dN/dS* (figure 2A, *dN* comparisons: *Q* < 0.05, 2/5; *Q* < 0.01, 2/5; *Q* < 0.001, 1/5; figure 2B, *dN/dS* comparisons*: Q* < 0.05, 2/4; *Q* < 0.001, 2/4). Genes with expression limited to the *P. falciparum* sporozoite stage had a median *dN*/*dS* value of 0.446, over twice that of all other genes with single-stage expression (median *dN/dS* = 0.204). We also observed elevations in gametocyte *dN* and *dN/dS* and ookinete *dN/dS* compared to the schizont stage (figure 2A, *Q* < 0.05, 3/3 comparisons). We saw no evidence that the life cycle stages varied in basal mutation rate, as *dS* levels were comparable across all gene sets (figure 2C). Rather, the higher sporozoite-stage *dN/dS* appears to be driven by *dN* increases (figure 2A). These results are consistent with work by Tebben *et al.,* who found higher mean *dN/dS* values in genes expressed in the sporozoite stage of the rodent parasite *P. berghei* [15].

### (f) Patterns of polymorphism vary across life stages

To examine patterns of polymorphism across genes expressed in single life cycle stages, we used data from the MalariaGen network documenting parasite variation within four geographically diverse populations [22]. We calculated gene-level diversity metrics including *π*_NS_, *π*_S_, *π*_NS_/*π*_S_ and Tajima’s *D* (electronic supplementary material, file S3). Although negative Tajima’s *D* estimates generally suggest predominant purifying selection, *P. falciparum* populations show a strong negative skew in *D* genome-wide [18,60,61]; we therefore interpret relative elevations in *D* as signals of increased drift.

**Figure 3.**
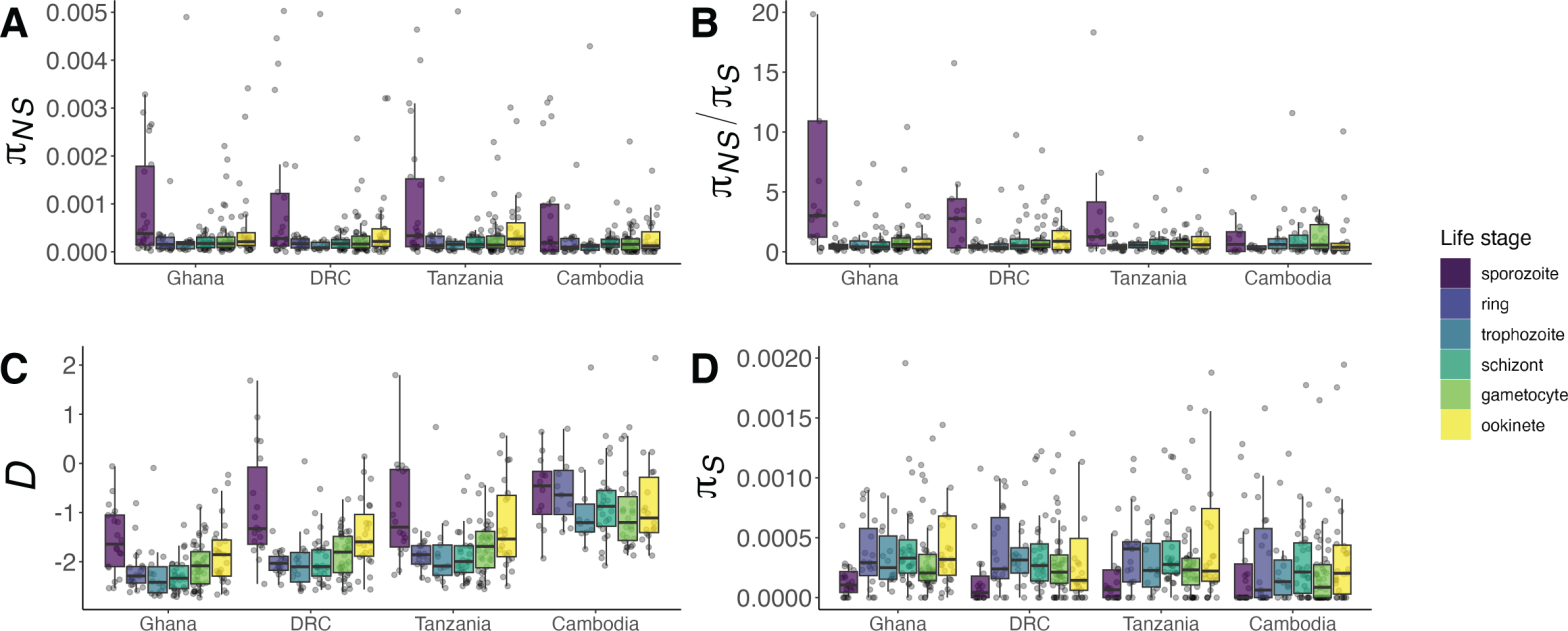
Gene-level population diversity statistics including (A) *π*_NS_ (2 outliers above y-axis limit not shown), (B) *π*_NS_/*π*_S_ (9 outliers above *y*-axis limit not shown and 105 infinite values not represented), (C) Tajima’s *D* and (D) *π*_S_ (22 outliers above *y*-axis limit not shown) across four geographically diverse parasite populations (*x*-axis). Estimates are shown for genes with single-stage expression and grouped by life stage, which is denoted by boxplot colour.

We used linear regression to assess the association between gene-level diversity estimates and categorical life stage, examining pairwise differences in mean estimates for each stage. To aggregate stage-level trends across all four separate populations, we used AW-Fisher *p*-value meta-analysis on the resulting population-level associations. We found increases in *π*_NS_ and *π*_NS_/*π*_S_ for genes only expressed in the sporozoite stage relative to those only expressed in four of the other five stages (figure 3A,B; electronic supplementary material, table S1, *Q* > 0.05 sporozoite–trophozoite *π*_NS_ comparison; *Q* < 0.01, sporozoite–ookinete *π*_NS_, *π*_NS_/*π*_S_ and sporozoite–trophozoite *π*_NS_/*π*_S_ comparison; *Q* < 0.001, all other comparisons). However, we did not generally see this trend for *π*_S_, a proxy for neutral sequence change, although we did observe an elevation of *π*_S_ in ookinete genes compared to sporozoite and gametocyte genes (figure 3D; electronic supplementary material, table S1, *Q <* 0.01, sporozoite–ookinete *π*_S_ comparison and *Q* < 0.001, gametocyte–ookinete *π*_S_ comparison). Genes expressed only in the sporozoite stage also showed higher Tajima’s *D* values than those expressed in four of the five alternative stages, while ookinete genes had higher *D* values than ring stage genes (figure 3C; electronic supplementary material, table S1, *Q* > 0.05, sporozoite–ookinete *D* comparison; *Q* < 0.05*,* sporozoite–trophozoite *D* comparison and ookinete-ring *D* comparison*; Q* < 0.001, all other comparisons). We obtain comparable results for sporozoite genes when comparing these estimates between our alternatively defined gene sets with single-stage expression (electronic supplementary material, figure S9, table S2). In these secondary gene sets, ookinete genes also show elevations in *π*_NS_/*π*_S_ (electronic supplementary material, table S2).

### (g) Population-level genetic differentiation varies across life stages

Next, we compared genetic polymorphism across parasite populations. We hypothesized that population differentiation may differ by stage of expression, which could be attributed to either geographically variable selection on stage-specific functions or reduced constraint (higher drift) operating at some life cycle stages. We estimated per-gene Hudson’s *F_ST_* in pairwise comparisons of four parasite populations sampled in Ghana, Tanzania, the Democratic Republic of the Congo and Cambodia (electronic supplementary material, file S3).

**Figure 4.**
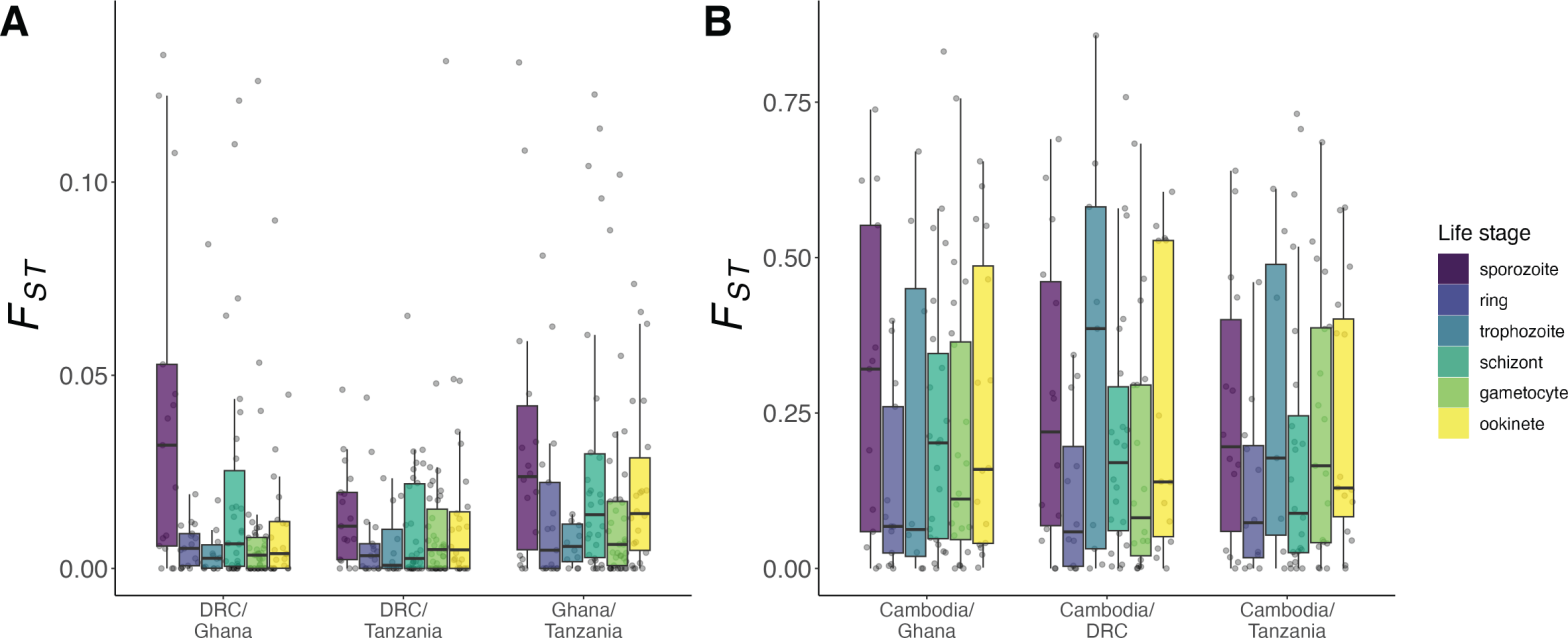
Hudson’s *F_ST_* estimates comparing *P. falciparum* populations, defined here at the country level, (A) within the African continent (4 outliers above y-axis limit not shown) and (B) between Africa and Asia. Sample sizes are 362, 136, 152 and 75 for Ghana, Tanzania, the Democratic Republic of the Congo and Cambodia, respectively.

Africa is the inferred point of origin for *P. falciparum*, with the parasite probably spreading to Southeast Asia 50 000 to 60 000 years ago [62]. We used linear regression to test for pairwise *F_ST_* differences between stages, again aggregating the results of population comparisons with AW-Fisher meta-analysis of *p*-values. We found that sporozoite stage genes had a higher *F*_ST_ than genes solely expressed in other stages except the schizont stage (figure 4; electronic supplementary material, table S3; *Q* < 0.05, 2/4 comparisons; *Q* < 0.001, 2/4 comparisons). These differences are most pronounced for the within-Africa comparisons given the higher connectivity between these populations and corresponding lower *F*_ST_ range for comparisons between them (figure 4A).

### (h) Weaker inferred negative selection in the sporozoite and ookinete stages

After finding that expression in different life stages may impact substitution rates across the *P. falciparum* genome, we examined the strength of selection in each stage. We inferred the distribution of fitness effects (DFE), or selection coefficients, on new coding substitutions in each gene set with DFE-alpha [50,51] and fastDFE [52]. DFE-alpha uses folded SFS data, which records minor allele frequencies, to estimate the deleterious DFE, given that most within-species polymorphism is expected to reduce fitness. This generates an expectation for the proportion of effectively neutral (slightly deleterious) substitutions such that excess between-species coding divergence is interpreted as adaptive. In contrast, fastDFE infers the deleterious and beneficial DFE jointly from unfolded SFS data, which incorporates the frequencies of derived (non-ancestral) alleles. We report summary statistics from these analyses, including, for DFE-alpha, the probability of fixation of a novel deleterious mutation (*Q*), the proportion of adaptive divergence (*α*) and the relative rate of adaptive substitution (*ω*) for each gene set. For fastDFE, we examine *α* along with the mean selection coefficients for deleterious and beneficial mutations, *S*_d_ and *S*_b_*.* Both *Q* and *S* estimates depend on population-scaled selection coefficients, although *Q* reflects these indirectly.

**Figure 5.**
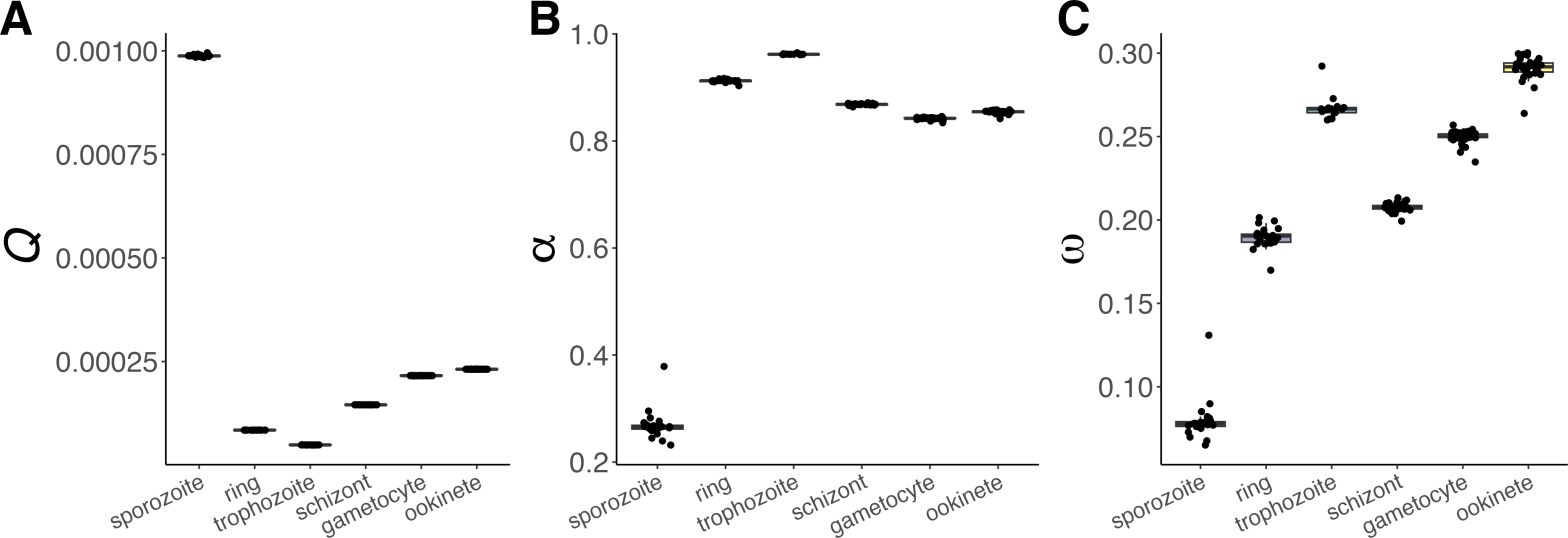
Jackknifed estimates of probability of fixation of a deleterious mutation (*Q*), proportion of adaptive substitution (*α*) and the relative rate of adaptive divergence (*ω*) estimated by DFE-alpha analysis of site frequency spectra and *P. falciparum–P. reichenowi* divergence estimates for non-synonymous (selected) and synonymous (neutral) SNPs in *P. falciparum* life stage-associated genes. All stage-stage pairwise comparisons of jackknifed estimates revealed significant differences by life stage (Bonferroni-adjusted Wilcoxon rank sum test *p* < 0.001).

Relying on a jackknifing approach to generate outlier-robust comparisons of gene sets with single-stage expression, we assessed *Q*, *α* and *ω* for each gene set category (figure 5). These estimates were all significantly different from each other across stages (*p* < 0.001, all comparisons). We show an increase in *Q* for the sporozoite stage (figure 5A; median *Q*_sporozoite_ = 9.88 × 10^−4^) relative to all other stages and particularly the blood stages (median *Q*_non-sporozoite_ = 2.16 × 10^−4^, median *Q*_ring | trophozoite | schizont_ = 8.45 × 10^−5^). These estimates imply that a novel deleterious mutation is over ten times more likely to fix if it arises in a gene expressed only in the sporozoite stage compared to genes expressed only in one of the blood stages. Genes expressed only in gametocytes or ookinetes are estimated to fix deleterious polymorphism over twice as often as genes expressed only in an asexual blood stage (median *Q*_ookinete_ = 2.31 × 10^−4^, median *Q*_gametocyte_ = 2.16 × 10^−4^). This is consistent with deleterious DFE inference in fastDFE, which estimates a practically 0 −*S*_d_ (4*N*_e_*s*) for sporozoite genes (electronic supplementary material, figure S10A, 9.99 × 10^−9^) compared to other stages (electronic supplementary material, figure S10A; median −*S*_d_ non-sporozoite = 7.67 × 10^3^). Interestingly, this technique also inferred the strongest negative selection in the gametocyte and schizont stages (−*S*_d_ = 10^5^*).* However, the latter estimates fell at the upper *S*_d_ bound and the former sporozoite model showed a high L1 norm, suggesting poor model fits.

Overall, the sporozoite gene set has proportionally fewer adaptive polymorphisms than the other gene sets (figure 5B; median *α*_sporozoite_ = 0.266, median *α*_non-sporozoite_ = 0.857). We obtain similar relative elevations in *Q* and reductions in *α* for the sporozoite gene set when we use FFD sites as neutral sites (electronic supplementary material, figure S11A, C, E), when we analyse *P. falciparum*–*P. praefalciparum* divergence (electronic supplementary material, S11B, D, F), when we use population data from the other African parasite populations (electronic supplementary material, figure S12A–D), or when we do not implement a demographic correction (electronic supplementary material, figure S13A,B). However, we find no significant differences in *α* estimates for any gene set with fastDFE inference from polymorphism data (bootstrapped 95% confidence intervals, electronic supplementary material, figure S10B). These models did not demonstrate significant beneficial fitness effects by likelihood ratio tests (electronic supplementary material, figure S14, Bonferroni-adjusted LRT *p* > 0.05), indicating that the inferred beneficial DFEs were poor fits to the data.

### (i) Stronger inferred directional selection in the ookinete and trophozoite stages

Unlike *dN/dS*, which does not distinguish between adaptive and non-adaptive coding changes, our *ω* estimates are adjusted to reveal underlying differences in selection efficacy. The sporozoite stage gene set had the lowest estimated rate of adaptive divergence (*ω*), while the ookinete and trophozoite sets had the highest (figure 5C). Considering *Q* and *ω* jointly, these results are consistent with stronger negative selection in ring and schizont stages. However, *ω* estimates were more sensitive to changes in analysis parameters and the exclusion of single genes than *Q* and *α* estimates (figure 5; electronic supplementary material, S11–S13).

When we only consider substitutions at FFD sites to be neutral (electronic supplementary material, figure S11A, C, E) or when we investigate divergence between *P. falciparum* and its closest known relative *P. praefalciparum* rather than *P. reichenowi* (electronic supplementary material, figure S11B, D, F), we observe similar *Q*, *α,* and *ω* rankings, with sporozoite genes maintaining the lowest proportion of adaptive mutations.

When the DFE estimation step relies on parasite population data from the DRC and Tanzania, the trophozoite *α* estimates remain the highest among the gene sets but decrease by about 8–10% (electronic supplementary material, figure S12D,E median *α*_trophozoite,DRC_ = 0.895, median *α*_trophozoite,Tanzania_ = 0.873) compared to the estimates using population data from Ghana (figure 5B; *α*_trophozoite,Ghana_ = 0.974). This analysis also ranks the sporozoite gene set lowest and the trophozoite gene set highest in terms of adaptive divergence, but assigns the sporozoite gene set implausible (negative) *α* and *ω* values, indicating that the model does not accurately adjust for the demography of these populations (electronic supplementary material, figure S12C–F).

## 4. Discussion

As the propensity of *P. falciparum* to evolve resistance to therapeutics poses an ongoing threat to malaria elimination efforts [14], evolutionary considerations have gained increased prominence in therapeutic development [63]. Vaccine trials evaluate construct diversity [64,65], drug studies dissect patterns of *in vitro* resistance evolution [14,63], and policy makers weigh the potential for opposing selection pressures with specific drug combinations [66]. Here, we foreground an additional consideration: variability in adaptive potential across the parasite life cycle may impact long-term therapeutic success, and may be particularly relevant for the selection of targets and the timing of interventions [14,67].

Dissecting life stage-specific patterns of adaptation by isolating them within genes expressed in single stages, we find that selection efficacy may be lowest in mosquito and transmission stages and the sporozoite stage in particular. This is consistent with the expectation that limited clonal competition, transmission bottlenecks and higher ploidy will reduce selection efficacy. If this explanation is correct, the blood stages are poised to respond more effectively to novel selection pressures than the mosquito and transmission stages. An important caveat is that we conduct our analysis and infer parameters relevant to drift and selection in the context of negative selection. Although we expect that reduced selection efficacy should weaken positive selection in tandem with negative selection by raising the drift barrier, our gene sets do not support inference of beneficial selection from polymorphism data alone (electronic supplementary material, figure S14), making it difficult to predict the ultimate consequences of elevated drift for adaptation to novel positive selection pressures. Overall, however, these results demonstrate that patterns of variation diverge across *P. falciparum* life stages, which calls for further investigations of this phenomenon.

We find population genetic signatures of elevated drift in genes that are expressed only in the sporozoite stage, including increased *π*_NS_ and *π*_NS_/*π*_S_ (figure 3). These signals demonstrate that nonsynonymous mutations accumulate to a greater extent in these genes, showing inheritance patterns consistent with genetic drift or positive selection. We further estimate that deleterious mutations are 10 times more likely to become fixed if they arise in genes expressed only in the sporozoite stage compared to genes expressed in one of the asexual blood stages (figure 5) and that deleterious coding mutations observed in this gene set may have a nearly neutral mean selection coefficient close to 0 (electronic supplementary material, figure S10).

Considered along with divergence data, these patterns suggest that sporozoite stage genes harbour proportionally fewer adaptive polymorphisms than blood stage genes. The latter show unusually high proportions of adaptive polymorphism close to 90%. While we implemented a demographic correction in our model to remove upward bias in *α*, these absolute estimates could reflect residual bias stemming from this simplified model. They could also reflect neutral departures of SFS data from standard expectations due to unique aspects of parasite life history, including selfing and unequal offspring numbers [68]. Finally, they might reflect limitations in the sampling of beneficial polymorphism due to gene set size, a problem which could be magnified during DFE inference from polymorphism data alone, which did not uncover statistically significant beneficial fitness effects (electronic supplementary material, figure S14). Nonetheless, work in other organisms has shown large variation in α estimates, which have ranged from 25% to 94% in *Drosophila* species alone [69,70], based on the estimation techniques used and genomic regions analysed [71]. High *α* values have also been observed in haploid pathogens and commensals [71,72], suggesting that the ploidy and life history characteristics of these organisms may enhance adaptation. Whether or not inflations beyond this range represent true elevations unique to *P. falciparum* biology or to the focal gene sets is unclear, but could represent an intriguing future research direction in light of the dually intensified drift and selection driven by the *Plasmodium* life cycle [18]. Importantly, we expect the relative ranks of stage-level α estimates to be robust to demographic biases that inflate α estimates similarly across the whole genome. To this end, we observed that upper and lower α rankings were stable when implementing the demographic model compared to the null baseline model of parasite demography (electronic supplementary material, figure S13).

We hypothesize that factors like reductions in clonal competition, bottlenecks imposed during transmission or lingering bi-allelic protein expression may contribute to drift-dominated evolutionary regimes on sporozoite-expressed genes. The parasite undergoes its highest number of mitotic divisions in the asexual blood stages, potentiating intense clonal interference unique to this period of exponential parasite growth. Mutations impacting fitness in other stages may arise in the asexual blood stages, drift in the absence of selection and become stochastically fixed during the subsequent human–mosquito transmission bottleneck. Finally, while the parasite undergoes nuclear meiotic division after forming a zygote (figure 1), there is some evidence for diploid protein expression continuing through the sporozoite stage [73]. This increased functional ploidy could mask deleterious alleles and reduce selection efficacy in these stages relative to stages with strictly haploid protein expression.

Overall, these population genetic signals are consistent with there being lower selection efficacy at the sporozoite, and to a lesser extent, ookinete and gametocyte stages. However, this pattern may also reflect differences in selection pressures across life stages. Sporozoites may encounter more diverse selection pressures within or between populations as they cross tissues and evade immune responses in both mosquito and human. This would be consistent with sporozoite genes’ elevated *F*_ST_, but our *ω* estimation does not support this hypothesis as we found that the sporozoite stage gene set did not have the highest inferred rate of adaptive substitution (figure 5C; electronic supplementary material, figures S11 E–F, S12 E–F and S13C). Alternatively, sporozoite stage genes may experience weaker purifying selection on coding regions. For example, protein structural features, such as intrinsically disordered regions, are enriched in sporozoite-stage genes [74] and may be more robust to coding change.

Other factors—including genetic draft, recombination rate variation and GC-biased gene conversion—also impact intra-genomic patterns of diversity and divergence. However, genes within each of our gene sets are distributed genome-wide and show no clear colocalization-based mechanism for any of these biases (electronic supplementary material, figure S15).

In addition to stage-limited effects, we find evidence that the totality of a gene’s expression profile is worth considering when assessing evolutionary trajectories. Across the *P. falciparum* genome, we find evidence that negative selection is stronger when genes are expressed in more stages of the parasite life cycle. We observe modest decreases in *dN*/*dS* with expression breadth, consistent with increased constraint and paralleling an analogous *dN*/*dS* trend in rodent *Plasmodium* species [15]. We also see a weak negative but not significant decrease in *π*_NS_/*π*_S_ with breadth in the largest parasite population. As stated by Tebben *et al.*, these trends could reflect the tendency of core functional genes to be broadly expressed across the parasite life cycle [11,15,75]. The upper three breadth categories (4–6) in our analysis were enriched for nuclear functions relative to the genomic background, which is consistent with this hypothesis (electronic supplementary material, figure S8). Higher breadth genes may also have increased overall expression, which is known to intensify negative selection against protein misfolding and explain large proportions of variation in *dN/dS* in other single-celled eukaryotes [35]. Finally, antagonistic pleiotropy across stages can also constrain adaptation to any single niche. It could contribute to the observed decrease in *dN*/*dS* with breadth, depending on its prevalence across the genome. Across the whole transcriptome, we found signals of inverse regulation of gene expression in more physiologically dissimilar stages (electronic supplementary material, figure S2). We speculate that these negative correlations reflect some compartmentalization of transcriptional architecture across the life cycle, consistent with the hypothesis that complex life cycles enable adaptation to distinct selective environments. This architecture likely reduces antagonistic pleiotropy across the genome.

The implications of our observations for different therapeutic selective pressures—such as both positive and negative selection imposed by a novel drug compared with diversifying or balancing selection imposed by a vaccine—warrant further investigation. Simulation studies, building off of those modelling the whole malaria life cycle [18] or modelling evolution across haploid–diploid life cycles [20] may be extended to identify specific drivers and predict outcomes of life stage-specific differences in organismic evolution.

## Data Availability

Data were derived from the following sources. *P. falciparum* genomic variation data: MalariaGEN Pf7 release [22]. Inclusion criteria for samples (electronic supplementary material, file S2) and variant sites in VCF data are described in text. *P. falciparum* single-cell sequencing data: Malaria Cell Atlas *P. falciparum* Smart-seq2 dataset [21], file pf-ss2-set1.zip. Microarray antigen reactivity data: electronic supplementary material, dataset S1 in [28]. Sporozoite gold standard dataset: electronic supplementary material, table S3 in [27]. *P. praefalciparum* and *P. reichenowi* reference DNA and amino acid sequences: NCBI Genbank and Refseq assemblies GCA_900095595.1 and GCF_001601855.1. *P. falciparum* 3D7 reference DNA and amino acid sequences: PlasmoDB release 66 files PlasmoDB-66_Pfalciparum3D7_AnnotatedCDSs.fasta and PlasmoDB-66_Pfalciparum3D7_AnnotatedProteins.fasta. *P. falciparum* 3D7 reference coordinates: PlasmoDB release 64 and 31 files PlasmoDB-64_Pfalciparum3D7.gff and PlasmoDB-31_Pfalciparum3D7.gff (converted to bed format), hosted by VEuPathDB, as described in [76]. No original data were generated in the preparation of this publication. Code supporting this article has been uploaded at Zenodo [77]. Supplementary material is available online [78].
